# Evaluation of Hepatitis A and B Seroprevalences in Health-Care Workers

**DOI:** 10.5152/eurasianjmed.2024.23110

**Published:** 2024-06-01

**Authors:** Elif Sedanur Utlu, Nihan Ak

**Affiliations:** 1 Department of Family Medicine, Erzurum City Hospital, Erzurum,Türkiye; 2 Department of Occupational Diseases, Erzurum City Hospital, Erzurum, Türkiye

**Keywords:** Hepatitis antibodies, hepatitis B virus infection, secondary immunization

## Abstract

**Background:**

Viral hepatitis is still a common and important public health problem for health-care workers around the world and in our country. This study was aimed at determining the immunity status of hepatitis B and hepatitis A in order to vaccinate non-immune workers.

**Methods:**

The population of this cross-sectional descriptive study, which was conducted retrospectively, consists of all health-care workers who applied to the Occupational Health and Safety Department of the hospital between September 1, 2022, and December 31, 2022, for periodic examination. The files of 1652 health-care workers were examined without selecting the sample and these individuals were included in the study. “Annex 2 form,” which was delivered to the employees, was used as a data source in the research. Statistical analyses were performed by Statistical Package for the Social Sciences Statistics software (SPSS Inc.; Chicago, IL, USA), version 15.0.

**Results:**

In our study, the immunity against hepatitis B in the 18-29 age group, women, singles, and university graduates was statistically significantly higher, and the immunity against hepatitis A of the participants aged 50 and over, men, and primary school graduates was statistically significant and was found to be significantly higher (*P *< .05).

**Conclusion:**

The fact that 90.2% of the participants were immune to hepatitis B and 85.0% of the participants were immune to hepatitis A seems to be related to the success of vaccination programs for health-care workers in our country. It is very important that the immunization status of health-care workers, who are in the high-risk group in terms of infectious diseases, is at the desired level.

**Main Points**

- It was found that anti-hepatitis B (HBs) positivity was 90.2%, anti-hepatitis A virus (HAV) IgG positivity was 85.0%, and hepatitis B surface antigen (HBsAg) positivity was 1.4% in the personnel participating in the study.
- Anti-HBs positivity was found to be significantly higher in women, and anti-HAV IgG positivity was found to be significantly higher in men (*P *< .05).
- While anti-HAV IgG positivity was 98.3% in primary school graduates, it was 80.1% in university or higher graduates.
- In those without chronic disease, anti-HBs positivity was 91.3% and anti-HAV IgG positivity was 84.5% (*P *< .05).
- According to age groups, anti-HBs positivity was highest in the 18-29 age group (94.1%), and anti-HAV IgG positivity was higher in the 40-49 age group (96.7%).

## Introduction

Viral hepatitis is still a common and important public health problem around the world and in our country. According to the 2019 data from the World Health Organization (WHO), 296 million people have chronic hepatitis B virus (HBV) infection; 1.5 million new cases are added to these numbers every year; and it is estimated that 1.1 million people die due to these infections and their effects, including liver cancer (hepatocellular carcinoma), cirrhosis, and other conditions caused by chronic viral hepatitis.^1^ The prevalence of hepatitis B surface antigen (HBsAg) positivity in Türkiye is 4.6% and it has been reported that approximately 3 million people have chronic hepatitis B infection, and our country is among the middle endemic regions with these data.^2^ The most common causes of chronic liver disease in our country are chronic viral hepatitis developed secondary to hepatitis B and hepatitis C virus infections.^2^ Therefore, a global action plan has been published by the WHO to prevent and control the spread of viral hepatitis.^3^ It has been called for a 90% reduction in new hepatitis B and hepatitis C cases, a 65% decrease in hepatitis B-related deaths, and a global elimination of viral hepatitis by the year 2030, and global targets were determined in this direction.^4^

Hepatitis B and C are transmitted in a parenteral way, through infected blood and body fluids, percutaneous and mucosal contact, sexual intercourse with an infected partner, and perinatally. Health-care workers are in a high-risk group for transmission of these viruses due to frequent contact with blood and blood products, infected body fluids, frequent exposure to needle sticks or stab wounds, and percutaneous and mucocutaneous injuries because of their profession. Studies have shown that contact frequency increased, especially in surgical departments working in operating rooms and delivery rooms and personnel working in emergency services.^5^

According to WHO data, it has been stated that more than 85 million health-care workers worldwide are injured by contaminated medical equipment.^6^ Studies are reporting that HBsAg positivity is 1.9-15.6%, and anti-HCV positivity is 0% to 2.1% among health-care workers in Türkiye.^7,8^ Infectious diseases have been accepted as occupational infectious diseases by WHO and ILO (International Labor Organization), and hepatitis B infection was defined as an occupational disease for health-care workers in 1992. In 1996, the Turkish Ministry of Health screened health-care personnel and vaccinated those who were not immunized or whose immunization was inadequate.^9^ To prevent these infections, it is necessary to organize education within the scope of health surveillance, such as employment examinations and periodic examinations, the use of individual protective equipment, and performing screenings and vaccinations. The transmission rate of hepatitis B infection has been reported as 2-40% in unvaccinated individuals after sharp and puncture wounds, and it has been shown that transmission can be prevented by 90%-95% with routine vaccination.^10^ Hepatitis A is most commonly transmitted by the fecal or oral route, while it can also be transmitted by sexual intercourse and the parenteral route, with a small probability.^11^ Although it is often subclinical in childhood, exposure to the virus may have a fulminant course in adulthood. Today, with the development of the socioeconomic levels of societies, the probability of encountering hepatitis A may shift to adult ages.^8^ Therefore, it is vital to give 2 doses of the hepatitis A vaccine to people who are not immune at 0 and 6 months.

Viral hepatitis can progress to cirrhosis, liver cancer, and liver failure and cause mortality and morbidity. For this reason, it is necessary to determine the hepatitis A and B immunity status of health-care workers, which are classified as very dangerous workplaces according to our legislation, to provide necessary training on viral hepatitis to seronegative employees, and to make vaccination plans. The aim of this study was to determine the immunization prevalence of health-care workers in a city hospital based on hepatitis A and hepatitis B serology results. Those with insufficient immunization status were educated on the importance of immunization against hepatitis A and B and directed to the vaccination unit by making a vaccination plan.

## Material and Methods

Health-care workers who applied to our hospital’s Occupational Health and Safety Department (OHSD) between September 1, 2022, and December 31, 2022, were included in this study. The population of this cross-sectional-descriptive study, which was conducted retrospectively, consists of all health-care workers who applied to the OHSD of the hospital between September 1, 2022, and December 31, 2022, for periodic examination.

The files of 1652 health-care workers were examined without selecting the sample, and these individuals were included in the study. “Annex 2 form,” which was delivered to the employees, was used as a data source in the research. This study was approved by Ethics committee of Atatürk University Clinical Research (Approval number: B.30.2.ATA.0.01.00/211, Date: 30.03.2023). We carried out the study in accordance with the principles of the Declaration of Helsinki. Informed consent was obtained from participants.

Statistical analyses were performed by Statistical Package for the Social Sciences Statistics software (SPSS Inc.; Chicago, IL, USA), version 15.0. Frequency, percentage, mean value ± standard deviation, and median (minimum and maximum) were used for descriptive statistics. A chi-square test was used for the analysis of categorical data. The statistical significance value was accepted as *P *< .05. Values with *P *= .000 are stated as *P *< .001. The dependent variables of the study were hepatitis B and A immune status. Independent variables are sociodemographic (age, gender, and educational status) data and characteristics related to working life (unit work, job).

The results were seropositivity for HAV and HBV antibody statuses. Serological markers were determined by the enzyme-linked immunosorbent assay (AxSYM Abbott, ARCHITECT i2000; Abbott, USA). Anti-HAV IgG, serum HBsAg, and anti-HBs antibody results were recorded. Absence of anti-HBc or HBsAg and anti-HBs antibody levels of 0-10 mIU/mL for HBV and absence of anti-IgG for HAV were considered to indicate seronegativity.

After determining the hepatitis A and B immunity levels of health-care workers, seronegative health-care workers were contacted by phone and asked to apply to the OHSD. Health-care workers who applied to the OHSD were informed that their immunization levels were insufficient. They were directed to the vaccination unit by making a vaccination plan according to the vaccination schedule.

## Results

A total of 1652 people participated in the study; 50.5% were women and 49.5% were men. The mean ± standard deviation age of the participants was 36.5 ± 9.7 years, and the median age was 34 (minimum: 21, maximum: 64). It was determined that 58.1% of the participants were university graduates or higher, 33.9% were high school graduates, and 68.7% were married. Nurses/midwives were 48.3% of the health-care workers, 18% were cleaning staff, 10.3% were medical secretaries, and doctors were <1%. It was observed that 28.1% of the participants worked in the wards, 17.4% in the intensive care unit, 12.4% in the operating room, and 4.5% in the emergency department. Also, 18.4% had a chronic disease, and 30.4% of them were smokers (presented in Tables 1 and 2). It was found that anti-HBs positivity was 90.2%, anti-HAV IgG positivity was 85.0%, and HbSAg positivity was 1.4% in the personnel participating in the study (Table 2).

Anti-HBs positivity was found to be significantly higher in women, and anti-HAV IgG positivity was found to be significantly higher in men (*P *< .05). While anti-HAV IgG positivity was 98.3% in primary school graduates, it was 80.1% in university or higher graduates. Anti-HAV IgG positivity was 88.3% in married people, and the difference was statistically significant (*P *< .05). In those without chronic disease, anti-HBs positivity was 91.3% and anti-HAV IgG positivity was 84.5% (*P *< .05). In smokers, anti-HBs and anti-HAV IgG positivity were found to be significantly higher. According to age groups, anti-HBs positivity was highest in the 18-29 age group (94.1%), and anti-HAV IgG positivity was higher in the 40-49 age group (96.7%). (presented in Tables 3 and 4).

## Discussion

In this study, 39.2% of the participants were 18-29 years old, 26.0% were 40-49 years old, and the mean age was 36.5 ± 9.7 years. Of the participants, 50.5% were women, 49.5% were men, 31.0% were single, 33.9% were high school graduates, and 58.1% were university graduates. In a study conducted with employees of a state hospital in Eskişehir, 35.5% of the participants were male, and the mean age was reported as 35.78 ± 8.76, similar to our study.^8^ In a similar study conducted in India, it was determined that 52.95% of the participants were female and 47.05% were male health-care workers.^12^

Similar to the literature, it is known that the frequency of female and male employees in health-care institutions is close to each other. In the study, 48.3% of the health-care workers were nurses, 18.0% were cleaning personnel, 10.3% were secretaries, and approximately 1% were doctors. Approximately one-third of the participants stated that they worked in clinics, 18.7% in intensive care, and 13.3% in the operating room. In a study conducted with employees of a public hospital, it was determined that 7% of the participants worked as doctors, 19.7% as nurses, 9.1% as technicians, and 19.4% as cleaning staff.^5^ In our study, it was determined that the physicians working in our hospital did not participate in the periodic examinations compared to the other studies in the literature. Also, 30.4% of the participants in this study stated that they smoked, and the average pack/year of smokers was determined to be 15.4 ± 10.6. In a study conducted with the employees of a hospital of a medical school in 2016, it was determined that 46.3% of the participants smoked. This result shows that smoking habit is quite common among health-care workers in our country.^13^

Health-care workers with chronic diseases comprised 18.4% of the participants, and the most common chronic diseases in this study were thyroid diseases, hypertension, diabetes, and asthma. Similar to the literature, the chronic diseases seen in health-care workers in this study are similar to the most common chronic diseases in the world.^14^

In this study, anti-HBs levels were found to be high in 90.2% of the health-care workers, and they were found to be immune to hepatitis B. In a similar study conducted in Konya, anti-HBs levels were found to be 10-100 mIU/mL in 14.5% of the participants and ≥100 mIU/mL in 54.3%.^13^ Similarly, in another study, the anti-HBs positivity level was found to be 86%.^8^ The fact that hepatitis B vaccination is included in the childhood routine vaccination program in our country and that hepatitis B vaccination is among the recommended vaccines for health-care professionals explains the high level of immunity in these studies. In the current study, 85% of the participants were found to be immune to hepatitis A. In another study conducted in our country, anti-HAV IgG positivity was found in 71.7% of the participants.^8^ In a study conducted in our country in 2022, it was reported that 90.3% of the participants had anti-HAV IgG positivity, similar to our study.^15^ This may be due to the increased awareness by health-care professionals of the risk that infections due to hepatitis A can progress fulminantly in older people and cause death, and the increased rates of vaccination due to this.

In this study, it was observed that the immunity status against hepatitis B of the 18-29 age group, women, singles, and university graduates was statistically significantly higher (*P *< .05). In a study conducted in Italy, the hepatitis B immunity levels of female health-care workers were found to be statistically significantly higher, similar to our study.^16^ This result may be due to the high level of awareness about the importance of vaccination in the prevention of infectious diseases in the young age group with a high level of education and women. In addition, as seen in the literature, the fact that nurses are mostly women in our country, this group has to apply invasive procedures to patients frequently, and the risk of sharp and stab wounds is quite common in this group, may explain why hepatitis B vaccination is more common in this group.^17^ According to the adult vaccination guidelines in our country, 3 doses of hepatitis B vaccination are recommended for these individuals in all health-care institutions since health-care workers are among the groups at risk.^18^

In this study, it was determined that the hepatitis B immunity levels of the participants with chronic diseases and smokers were lower, and the difference was statistically significant (*P *< .05). Although health-care professionals with chronic diseases and smokers are mostly from older age groups, this may be related to the fact that these individuals were born before 1998, when vaccination was added to the routine vaccination program in our country. In this study, it was observed that the immunity status against hepatitis A of the participants aged 50 and older, males, and primary school graduates was statistically significantly higher (*P *< .05). Since hepatitis A infection is transmitted by the fecal–oral route, this infection is usually had in childhood in our country, but the infection can be fatal in elderly individuals. Therefore, the higher immunity level in elderly individuals and those with lower educational status in our study may be related to the fact that they had this infection in childhood.

The study’s strengths lie in demonstrating the hepatitis A and B seroprevalence among health-care workers at our hospital, thereby enabling the determination of the need for immunization. However, the study’s limitations arise from the low participation rate of health-care workers during periodic screenings.

As a result, in this study, the hepatitis A and B seropositivity of health-care workers was found to be quite high. This may be due to the increased level of health literacy and awareness of health-care workers compared to society. In addition to the success of vaccination programs in our country, issues that have priority for protection, such as personal hygiene education and the importance of using personal protective equipment while in contact with the patient, should be emphasized in all health-care institutions.

## Figures and Tables

**Table 1.** Participants’ Descriptive Characteristics

	n	(%)*
**Age groups (n = 1652)**		
18-29 years	647	39.2
30-39 years	368	22.3
40-49 years	429	26.0
50 years and older	208	12.5
**Gender (n = 1652)**		
Female	835	50.5
Male	817	49.5
**Marital status (n = 1652)**		
Single	512	31.0
Married	1135	68.7
Divorced	5	0.3
**Educational status (n = 1652)**		
Primary school	59	3.6
Secondary school	73	4.4
High school	560	33.9
Undergraduate degree	960	58.1
**Title (n = 1652)**		
Nurse	798	48.3
Cleaning staff	298	18.0
Secretary	170	10.3
Physician	15	0.9
Other**	371	22.5
**Departments (n = 1652)**		
Clinic	496	30.1
Intensive care	308	18.7
Operation room	219	13.3
Policlinic	153	9.2
Emergency department	78	4.7
Other***	398	24.0
**Smoking status (n = 1652)**		
Yes	502	30.4
No	1028	62.2
Quit smoking	122	7.4

*Column percentage.

**The most frequent responses in this group were “medical officer,” “technical staff,” and “security.”

***The most frequent responses in this group were “laboratory” and “technical unit.”

**Table 2.** Participants’ Health Status Related Characteristics

	n	(%)*
**Presence of chronic diseases (n = 1652)**		
** **Yes**	304	18.4
No	1348	81.6
**Status of surgery (n = 1652)**		
** **Yes***	642	38.9
No	1010	61.1
**Hepatitis B immunity (n = 1652)**		
** **Yes	1490	90.2
No	162	9.8
**Hepatitis A immunity (n = 1652)**		
** **Yes	1404	85.0
No	248	15.0
**Hepatitis B carrier (n = 1652)**		
Yes	23	1.4
No	1629	98.6

*Column percentage.

**The most frequent responses in this group were “thyroid diseases,” “hypertension,” “diabetes,” and “asthma.”

***The most frequent responses in this group were “cesarean section,” “ear, nose, and throat surgery,” and “hernia surgery.”

**Table 3.** Descriptive Characteristics of the Participants and their Hepatitis B Immune Status

	Hepatitis B Immunity			
	Immune		Not Immune	
	Number	(%)*	Number	(%)*
**Age groups (n = 1652)**				
** **18-29 years	609	94.1	38	5.9
30-39 years	325	88.3	43	11.7
40-49 years	382	89.0	47	11.0
50 years and older	174	83.7	34	16.3
*P* < .001^†^				
**Gender (n = 1652)**				
** **Female	777	93.1	58	6.9
Male	713	87.3	104	12.7
*P* < .001^†^				
				
**Educational status (n = 1652)**				
** **Primary school	54	91.5	5	8.5
Secondary school	59	80.8	14	19.2
Vocational school	477	85.2	83	14.8
Undergraduate degree	900	93.8	60	6.3
*P* < .001^†^				
**Marital status (n = 1652)**				
** **Single	481	93.9	31	6.1
Married	1006	88.6	129	11.4
Divorced	3	60.0	2	40.0
*P* < .001^†^				
**Presence of chronic diseases (n = 1652)**				
				
Yes	259	85.2	45	14.8
No	1231	91.3	117	8.7
*P* = .001^†^				
**Status of surgery (n = 1652)**				
** **Yes	580	90.3	62	9.7
No	910	90.1	100	9.9
*P* = .871				
**Smoking status (n = 1652)**				
Yes	432	86.1	70	13.9
No	944	91.8	84	8.2
Quit smoking	114	93.4	8	6.6
*P* = .001^†^				

*Row percentage.

**Chi-square test was applied.

^†^Statistically significant.

**Table 4.** Descriptive Characteristics of the Participants and Their Hepatitis A Immune Status

	Hepatitis A Immunity			
	Immune		Not Immune	
	Number	(%)*	Number	(%)*
**Age groups (n = 1652)**				
** **18-29 years	469	72.5	178	27.5
30-39 years	319	86.7	49	13.3
40-49 years	415	96.7	14	3.3
50 years and older	201	96.6	7	3.4
*P* < .001^†^				
**Gender (n = 1652)**				
** **Female	669	80.1	166	19.9
Male	735	90.0	82	10.0
*P* < .001^†^				
**Educational status (n = 1652)**				
** **Primary school	58	98.3	1	1.7
Secondary school	71	97.3	2	2.7
Vocational school	506	90.4	54	9.6
Undergraduate degree	769	80.1	191	19.9
*P* < .001^†^				
**Marital status (n = 1652)**				
** **Single	398	77.7	114	22.3
Married	1002	88.3	133	11.7
Divorced	4	80.0	1	20.0
*P* < .001^†^				
**Presence of chronic diseases (n = 1652)**				
Yes	265	87.2	39	12.8
No	1139	84.5	209	15.5
p = .238				
**Status of surgery (n = 1652)**				
** **Yes	554	86.3	88	13.7
No	850	84.2	160	15.8
*P* = .236				
**Smoking status (n = 1652)**				
Yes	439	87.5	63	12.5
No	854	83.1	174	16.9
Quit smoking	111	91.0	11	9.0
*P* = .012†				

*Row percentage.

**Chi-square test was applied.

^†^Statistically significant.
